# Comparing the efficacy and safety of nafamostat mesylate versus citrate for anticoagulation in continuous renal replacement therapy: a systematic review and meta-analysis

**DOI:** 10.3389/fmed.2026.1831023

**Published:** 2026-07-06

**Authors:** Zhao Hua Zou, Ji Quan Zhang, Pei Jun Xiang, Xing Chen, Wei Qing

**Affiliations:** 1 Department of Nephrology, Deyang People’s Hospital, Deyang, China; 2 Anaesthesia and Surgery Centre, Deyang People’s Hospital, Deyang, China; 3 Department of Nursing, Aobaoka Hospital, Osakasayama, Japan

**Keywords:** anticoagulation, citrate, continuous renal replacement therapy, meta-analysis, nafamostat mesylate

## Abstract

**Background:**

Anticoagulation is pivotal to the successful implementation of continuous renal replacement therapy (CRRT). Systematic comparative studies between nafamostat mesylate (NM) and citrate are scarce. There is currently a lack of evidence-based support for clinical practice.

**Objective:**

To systematically compare the efficacy and safety of NM versus citrate for anticoagulation in CRRT.

**Methods:**

We performed a comprehensive literature search in PubMed, Web of Science, Embase, Cochrane Library, China National Knowledge Infrastructure (CNKI), Wanfang Database (Wanfang), China Science and Technology Journal Database (VIP), and China Biological Medicine Database (CBM). Two researchers conducted literature screening, data extraction, and quality assessment using standardized procedures and double-blinded methods. Statistical analyses were performed using Review Manager V.5.4 and STATA 15.1. Statistical heterogeneity among studies was quantified using the Chi-square and I-square tests, and publication bias was evaluated using Egger’s test and funnel plots.

**Results:**

A total of 18 studies involving 2,247 CRRT patient episodes were included. The meta-analysis showed no significant differences in filter lifespan or clotting events between NM and citrate (MD = −0.11, 95%CI: −1.87 to 1.65, *p* = 0.90; RR = 0.63, 95%CI: 0.25–1.59, *p* = 0.33). NM reduced the risk of bleeding events compared with citrate (RR = 0.54, 95%CI: 0.36–0.82, *p* = 0.003), subgroup analyses showed that NM was associated with a lower risk of bleeding events in high-risk bleeding, low-dose NM, and randomized controlled trials (RCTs) subgroups (RR = 0.47, 95%CI: 0.25–0.88, *p* = 0.02; RR = 0.39, 95%CI: 0.23–0.66, *p* < 0.001; RR = 0.21, 95%CI: 0.07–0.66, *p* = 0.007). No significant differences were found between NM and citrate in platelet (PLT), activated partial thromboplastin time (APTT), or prothrombin time (PT) (MD = 0.88, 95%CI: −5.83 to 7.59, *p* = 0.80; MD = −0.42, 95%CI: −2.74 to 1.91, *p* = 0.73; MD =-0.38, 95%CI: −1.45 to 0.68, *p* = 0.48).

**Conclusion:**

NM suggests anticoagulant efficacy comparable to citrate in CRRT, with a lower risk of bleeding, particularly in high bleeding risk situations, among patients receiving low-dose NM. For CRRT patients with contraindications to citrate or high bleeding risk, NM may be an anticoagulant alternative that combines efficacy with safety. The overall certainty of the evidence from this study is low to very low; further multi-center, large-sample, high-quality RCTs are required to validate these findings.

**Systematic review registration:**

https://www.crd.york.ac.uk/PROSPERO/view/CRD420251182609, identifier CRD420251182609.

## 1 Introduction

Continuous renal replacement therapy (CRRT) is an extracorporeal blood purification technique. It is widely employed in the emergency management of critically ill patients with acute kidney injury and multiple organ dysfunction. More than 75% of patients receive CRRT as the initial renal replacement therapy, and its successful implementation depends on safe and effective anticoagulation strategies (1). Unfractionated heparin (UFH) was once the predominant anticoagulant in CRRT, but its high bleeding risk and potential for heparin-induced thrombocytopenia have limited its clinical use. The Kidney Disease: Improving Global Outcomes (KDIGO) clinical practice guidelines (2) recommend prioritizing regional citrate anticoagulation (RCA) over heparin in CRRT therapy. However, for patients with contraindications to citrate, such as concomitant liver failure, shock, hypoxaemia, or acid-base imbalance (3), clinicians are often compelled to adopt a non-anticoagulation (NA) strategy. This approach can lead to shorter filter lifespan, reduced solute clearance efficiency, increased nursing workload and treatment costs, and potentially elevated mortality rates (4).

Nafamostat mesylate (NM) is a synthetic serine protease inhibitor that exerts anticoagulant effects by inhibiting key coagulation factors. This drug undergoes rapid metabolism via both hepatic and blood pathways, exhibiting an extremely short half-life of only 5–8 min (5, 6). It exerts minimal effects on *in vivo* hemostasis with low bleeding risk, establishing itself as a novel regional anticoagulant for extracorporeal circulation. It may serve as an alternative to RCA in CRRT. NM is widely used across Asian countries, particularly in Japan, where it is the most commonly used anticoagulant in CRRT for critically ill patients, with a utilization rate of up to 84.9%. It is especially suitable for patients with high bleeding risk or active bleeding (7).

Three systematic reviews have summarized the current status of NM in CRRT anticoagulation (8–10). Zhang et al. (8) included 4 studies comparing NM with NA, demonstrating that NM significantly extended filter lifespan without increasing bleeding risk. Lin et al. (9) compared NM with low-molecular-weight heparin (LMWH) and found a lower bleeding risk with NM. However, only one study compared NM with citrate for bleeding incidence and showed no statistically significant difference. Liu et al. (10) compared NM with citrate using only 3 direct-comparison studies; the small sample size yielded insufficient statistical power to draw definitive conclusions about filter lifespan or bleeding risk, thereby failing to provide precise clinical guidance.

Given the current insufficient evidence comparing NM with citrate, this study aims to systematically synthesize existing evidence through an updated, more comprehensive systematic review and meta-analysis. It will compare the efficacy and safety of the two anticoagulation regimens in CRRT, identify specific populations that may benefit through subgroup analysis, and provide more reliable evidence-based guidance for clinical decision-making.

## 2 Methods

### 2.1 Design

This study was completed using the Preferred Reporting Items for Systematic Reviews and Meta-Analyses (PRISMA) guidelines(11) (Supplementary Document 1). It was registered in the International Prospective Register of Systematic Review (PROSPERO): CRD420251182609.

### 2.2 Eligibility criteria

According to PICOS (Population, Intervention, Comparison, Outcomes, and Study design) principles (12), we determine the following inclusion criteria: (1) The population was critically ill patients receiving CRRT; (2) Intervention group receiving NM; (3) Control group receiving citrate; (4) Outcome measures were filter lifespan, bleeding events, clotting events, or coagulation function indicators, included studies must report at least one of these; (5) Study designs were randomized controlled trials (RCTs), quasi-experimental studies, prospective cohort studies, retrospective cohort studies, or case-control studies. Exclusion criteria: (1) without available full text; (2) review; (3) case report; (4) conference or letter; (5) not published in English or Chinese.

### 2.3 Search strategy

We searched the literature in 8 databases, including PubMed, Web of Science, Embase, Cochrane Library, China National Knowledge Infrastructure (CNKI), Wanfang Database (Wanfang), China Science and Technology Journal Database (VIP), and China Biological Medicine Database (CBM). All publications were indexed from the databases’ inception to December 2025. We also reviewed references for relevant studies. Combinations of controlled vocabulary using Medical Subject Headings (MeSH) terms and free-text terms. The search terms included nafamostat, nafamostat mesylate, nafamostat mesilate, nafamostat mediate, NM, nafamostat dimethanesulfonate, nafamostat dihydrochloride, futhan, 6’-amidino-2-naphthyl 4-guanidinobenzoate, FUT-175, CKD-314, continuous renal replacement therapy, CRRT, continuous kidney replacement therapy, CKRT, blood purification therapy, hemopurification, continuous venovenous hemofiltration, CVVH, continuous venovenous hemodialysis, CVVHD, continuous venovenous hemodiafiltration, CVVHDF, slow continuous ultrafiltration, SCUF. Boolean operators “OR, AND” were used for the search (Supplementary Document 2).

### 2.4 Study selection and data extraction

Two researchers independently performed the literature search, screening titles and abstracts, conducting full-text reviews, assessing study quality, and extracting data. Any disagreement was resolved by discussion until a consensus was reached or by consulting a third researcher. The extracted data included the first author, publication year, country, study design, sample size, patient type, nafamostat mesylate regimen, citrate regimen, and outcomes. Bleeding events were defined as new or worsening bleeding during CRRT, requiring red blood cell transfusion, or a decrease in hemoglobin level of ≥ 2 g/dL, or clinical manifestations such as gum bleeding, skin and mucosal bleeding, gastrointestinal bleeding, intracranial hemorrhage, and hematuria.

### 2.5 Quality assessment of the study

Two researchers assessed the risk of bias. They used version 2 of the Cochrane Risk of Bias tool (ROB 2) for RCTs (13) and the Risk Of Bias In Non-randomized Studies-of Interventions (ROBINS-I) for observational studies (14). The ROB 2 included the randomization process, deviation from the intended intervention, missing outcome data, outcome measures, selection of the reported result, and overall, each indicator contains 3 levels: low-risk, unclear, and high-risk. The ROBINS-I included confounding, selection of participants, intervention, deviations from intended interventions, missing data, measurement of outcomes, selection of the reported result, and overall, each indicator contains 5 levels: low-risk, moderate-risk, serious-risk, critical-risk, and no information. Any disagreements were resolved by consensus or through third-party adjudication.

### 2.6 Data synthesis and analysis

For dichotomous outcomes, pooled estimates were expressed as relative risk (RR) with 95% confidence intervals (95%CI). Continuous data were pooled as mean differences (MD) with 95%CI when the outcome measurement methods and units were identical across studies; otherwise, standardized mean differences (SMD) were used. The median and interquartile range were converted to the mean and standard deviation using the formulae provided by Wan et al. (15) and Luo et al. (16). Statistical heterogeneity among studies was quantified using the Chi-square and I-square (I^2^) tests. If *p*<0.10 or *I*^2^>50%, significant heterogeneity was observed among studies; the random-effects model was used to combine the data. Otherwise, a fixed-effects model was used. We used visual inspection of funnel plots and Egger’s test to assess publication bias (17). We conducted a sensitivity analysis to assess the robustness of the meta-analysis models. Statistical analyses were performed using Review Manager V.5.4 and STATA 15.1.

### 2.7 Confidence in evidence

We evaluated the confidence in the evidence using the Grading of Recommendations Assessment, Development, and Evaluation (GRADE) approach (18), which classified evidence as high, moderate, low, or very low based on factors such as risk of bias, inconsistency, indirectness, imprecision, and publication bias. Any disagreements were resolved by consensus or through third-party adjudication.

## 3 Results

### 3.1 Search outcome

**FIGURE 1:**
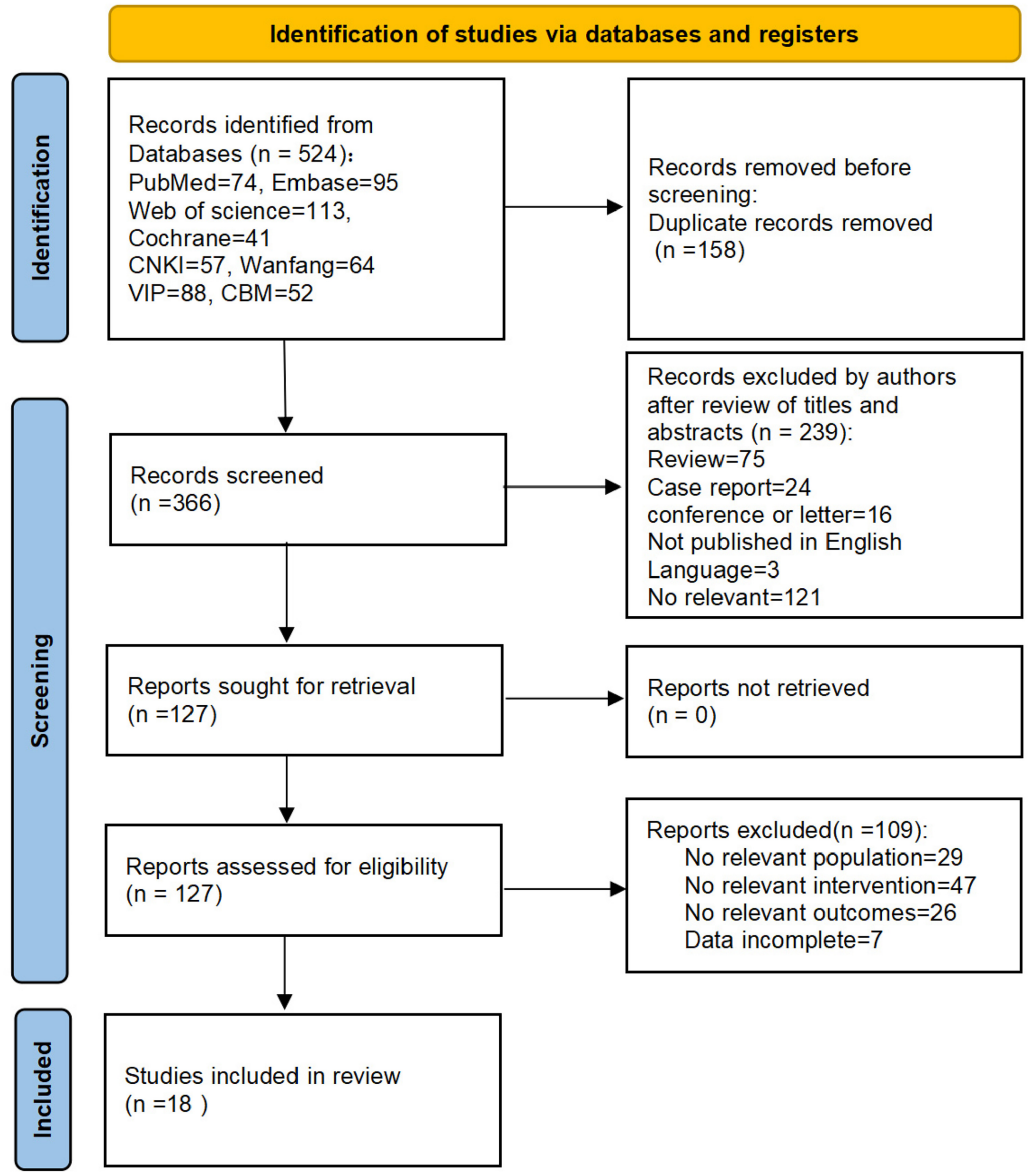
PRISMA flow diagram regarding article selection for meta-analysis.

A total of 524 studies were identified from eight databases. Using NoteExpress 3.5 software, 158 duplicate studies were removed. After reviewing titles and abstracts, 239 studies were excluded. After reading the full texts, a further 109 studies were excluded. Finally, 18 studies were included in the systematic review and meta-analysis. The flow diagram of search and selection is detailed in Figure 1.

### 3.2 Study characteristics

A total of 18 studies involving 2,247 CRRT patient episodes were included in this study. Among these, 5 studies were RCTs, 12 were cohort studies, and 1 was a case-control study. 7 studies were in English, while 11 studies were in Chinese. 988 CRRT patient episodes were in the intervention group receiving NM, and 1,259 were in the control group receiving citrate. The basic characteristics of the included studies are detailed in Table 1.

**TABLE 1:** Basic characteristics of the included studies.

Study	Country	Design	Sample, n(N/C)	Patient type	CRRT regimen	NM group	Citrate group	Outcomes
Chen et al. (19)	China	RCS	25/25	Patients receiving CRRT at high risk of bleeding	Machine: Baxter, modality: CRRT, blood flow: 150–200 mL/min, replacement solution: 1.5–2.5 L/h	Use saline containing 20 mg NM to flush the circuit and filter. NM administered via infusion pump at a rate of 10–50 mg/h.	4% citrate was infused at a rate of 3.3 mmol/L, and the 10% calcium chloride at a rate of 1.7 mmol/L.	Filter lifespan, bleeding events, PLT, APTT
He and Zhang (20)	China	RCS	17/15	Patients with acute liver failure receiving CRRT	Machine: Prismaflex (Baxter)/multiFiltrate (Fresenius), modality: CVVHDF	Use saline containing 20 mg NM to prime the circuit. The initial dose of NM was 0.1–0.5 mg/kg, and the maintenance dose was 0.1–0.5 mg/kg/h.	The initial 4% citrate flow rate (ml/h) was calculated as 1.5 × the blood flow rate (ml/min).	Filter lifespan, bleeding events
Jiang et al. (21)	China	RCT	54/54	Patients with acute kidney injury receiving CRRT	Machine: multiFiltrate(Fresenius), filter: AV1000S, modality: CVVHDF, blood flow: 150–180 mL/min, calcium-free replacement solution: 2-4 L/h, prescribed dose: 50-300 mL/h	Use saline containing 20 mg NM to flush the circuit and filter. NM administered via infusion pump at a rate of 20–30 mg/h.	4% citrate was infused at a rate of 200 mL/h, and the 10% calcium gluconate was supplemented.	Filter lifespan, clotting events, bleeding events, PLT, APTT, PT
Lan and Xu (22)	China	RCT	38/38	Patients with acute kidney injury receiving CRRT at high risk of bleeding	Modality: CRRT, blood flow: 150–200 mL/min, prescribed dose: 30–35 mL/(kg⋅h)	Use saline containing 20 mg NM to flush the circuit. The initial dose of NM was 0.4 mg/kg, and the maintenance dose was 0.4 mg/kg/h.	The 4% citrate flow rate was calculated as 1.2–1.5 × the blood flow rate.	Filter lifespan, bleeding events, PLT, APTT, PT
Liu et al. (23)	China	RCS	24/22	Patients receiving CRRT at high risk of bleeding	Machine: Prismaflex(Baxter), filter: ST100, modality: CRRT, replacement solution: 3L-4 L/h	Use saline containing 20 mg NM to fill the hemodialysis circuit. NM at a dose of 20–50 mg/h through an anticoagulant infusion line continuously.	4% citrate was connected to the arterial end of the hemofilter line through an infusion pump.	Filter lifespan, PLT, APTT, PT
Lu et al. (24)	China	RCS	10/10	Patients receiving CRRT at high risk of bleeding	Modality: CVVH	NM administered via infusion pump at a rate of 20 mg/h.	4% citrate was infused via the arterial end of the extracorporeal circuit, and the calcium-containing replacement solution was supplemented.	Filter lifespan, bleeding events
Miyaji et al. (25)	Japan	RCS	80/78	Patients receiving CRRT	Machine:Prismaflex (Baxter)/TR55X (Toray), filter: HF series/M series/UTseries, modality: CRRT	NM was given as a pre-filter into the CRRT circuit, with starting doses of 1 mg/kg, followed by a 1 mg/kg/h bolus.	4% citrate was accomplished via the arterial limb of the CRRT circuit, and the calcium infusion was supplemented.	Filter lifespan, clotting events, bleeding events
Niu et al. (26)	China	RCS	33/27	Patients with chronic kidney disease receiving CRRT at high risk of bleeding	Modality: CRRT	Pre-flush with NM at a dose of 0.1 mg/kg. NM administered via infusion pump at a rate of 0.5 mg/kg/h.	4% citrate was infused at a rate of 180 mL/h, and the 10% calcium gluconate was supplemented.	Filter lifespan, bleeding events, APTT, PT
Song et al. (27)	China	RCT	39/36	Patients with coagulation dysfunction receiving CRRT	Machine: Prismaflex(Baxter), filter: ST150, modality: CVVHDF, blood flow:150–200 mL/min, prescribed dose: 30-35 mL/(kg⋅h)	Use saline containing 5000U heparin to flush the circuit and filter. The initial dose of NM is 0.4 mg/kg, and maintain a dose of 0.4 mg/kg/h.	4% citrate was connected to the arterial end of the hemofilter line, and the calcium-containing replacement solution was supplemented.	Filter lifespan, PLT, APTT, PT
Song et al. (28)	China	RCS	32/47	Patients receiving CRRT at high risk of bleeding	Machine: Prismaflex(Baxter), filter: M100, modality: CVVH/CVVHDF, blood flow:150–200 mL/min, prescribed dose: 25–35 mL/(kg⋅h)	Use saline containing NM to flush the hemodialysis circuit. NM was given at an initial dose of 20–30 mg and a maintenance dose of 10–30 mg/h.	4% citrate was infused at a rate of 200 mL/h, and 10% calcium gluconate was infused via the central vein or the return end of the extracorporeal circuit.	Filter lifespan, bleeding events, PLT, APTT, PT
Tang et al. (29)	China	RCS	21/22	Patients with chronic kidney disease receiving CRRT at high risk of bleeding	Machine: Baxter, filter: 17R, modality: CVVHDF, blood flow: 180–240 mL/min, prescribed dose: 20–30 mL/(kg⋅h)	Use saline containing 20 mg NM to flush the circuit and filter. NM administered via infusion pump at a rate of 35–45 mg/h.	4% citrate was infused via the arterial end of the extracorporeal circuit, and the calcium chloride was supplemented.	PLT, APTT
Wang et al. (30)	China	RCT	40/40	Patients receiving CRRT	Machine: Baxter, modality: CVVH	Use saline containing 20 mg NM to flush the circuit and filter. NM administered via infusion pump at a rate of 30 mg/h.	4% citrate was infused at a rate of 200 mL/h.	bleeding events, APTT
Xiao et al. (31)	China	PCS	40/57	Patients receiving CRRT	Machine: Nipro, filter: 19U, modality: CVVH/CVVHD/CVVHDF, replacement solution: 2 L/h	The initial dose of NM was 10 mg, and the maintenance dose was 10–25 mg/h.	4% citrate was infused at a rate of 200–250 mL/h, and the 3% calcium chloride at a rate of 8–10 mL/h.	Filter lifespan, clotting events
Yu (32)	China	RCT	43/43	Patients receiving CRRT at high risk of bleeding	Modality: CRRT, blood flow: 150–300 mL/min	Use saline containing 20 mg NM to flush the circuit and filter. NM administered via infusion pump at a rate of 25 mg/h.	The 4% citrate flow rate was calculated as 2 × the blood flow rate, and the calcium-containing replacement solution was supplemented.	Filter lifespan, clotting events, bleeding events, PLT, APTT, PT
Yue et al. (33)	China	CCS	128/86	Patients with severe burns receiving CRRT	Machine: Fresenius, filter: AV600S, modality: CVVH/CVVHDF	NM administered via infusion pump at an average rate of 24 mg/h.	4% citrate was infused at an average rate of 217 mL/h.	Filter lifespan
Zeng et al. (34)	China	RCS	31/50	Patients receiving CRRT	Machine: Baxter/Fresenius, filter: ST150/M150/oXiris/AV1000S, modality: CVVH/CVVHD/CVVHDF, blood flow: 100–220 mL/min	Circuits were pre-flushed with NM. NM dosing started between 2 and 40 mg/h and was adjusted based on post-filter.	4% citrate was infused via the arterial end of the extracorporeal circuit.	Filter lifespan, bleeding events
Zhang and Li (35)	China	RCS	22/126	Patients receiving CRRT	Machine: Prisma flex(Baxter), modality: CVVH, blood flow: 130 mL/min	Use saline containing 20 mg NM to flush the circuit and filter. NM administered via infusion pump at a rate of 20–50 mg/h.	4% citrate was infused at a rate of 160–180 mL/h, and the 10% calcium gluconate at a rate of 10 mL/h.	Filter lifespan, clotting events, bleeding events, PLT, APTT, PT
Zhao et al. (36)	China	RCS	31/36	Patients with acute kidney injury receiving CRRT	Filter: AV600S, modality: CRRT	Use saline containing 20 mg NM to flush the circuit and filter. NM administered via infusion pump at a rate of 20–50 mg/h.	4% citrate was infused via the arterial end of the extracorporeal circuit.	Filter lifespan

RCT, randomized controlled trial; RCS, retrospective cohort study; PCS, prospective cohort study; CCS, case–control study; N, nafamostat mesylate group; C, citrate group; CRRT, continuous renal replacement therapy; CVVH, continuous venovenous hemofiltration; CVVHD, continuous venovenous hemodialysis; CVVHDF, continuous venovenous hemodiafiltration; NM, nafamostat mesylate; PLT, platelet; APTT, activated partial thromboplastin time; PT, prothrombin time.

### 3.3 Risk of bias

**FIGURE 2:**
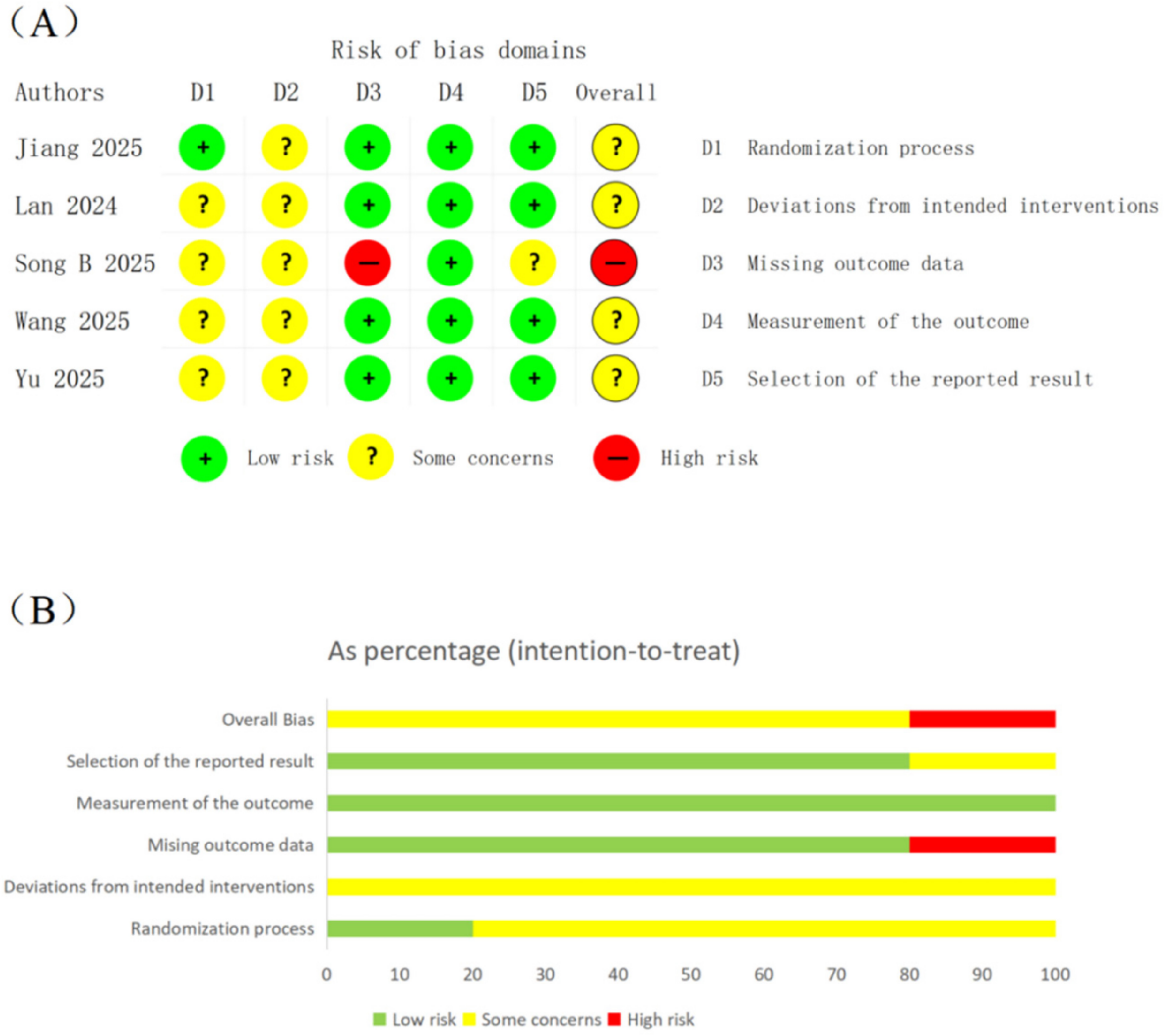
**(A)** Assessments of risk of bias for the included studies. **(B)** A summary of the risk of bias assessments for the included studies.

We used the ROB 2 tool to assess the risk of bias for 5 RCTs. 4 studies showed some risk of bias, and 1 study had a high risk of bias. Of these, 3 studies reported detailed randomization methods, and 1 described the allocation concealment process. No studies reported whether a blinding method was used for participants, interveners, or outcome assessors. The results of the risk of bias are detailed in Figure 2. We also used the ROBINS-I tool to assess the risk of bias for 13 observational studies. 7 studies had a moderate risk of bias, and 6 studies had a serious risk of bias. The quality assessment results are detailed in Supplementary Document 3.

### 3.4 Outcomes and effect sizes

#### 3.4.1 Filter lifespan

**FIGURE 3:**
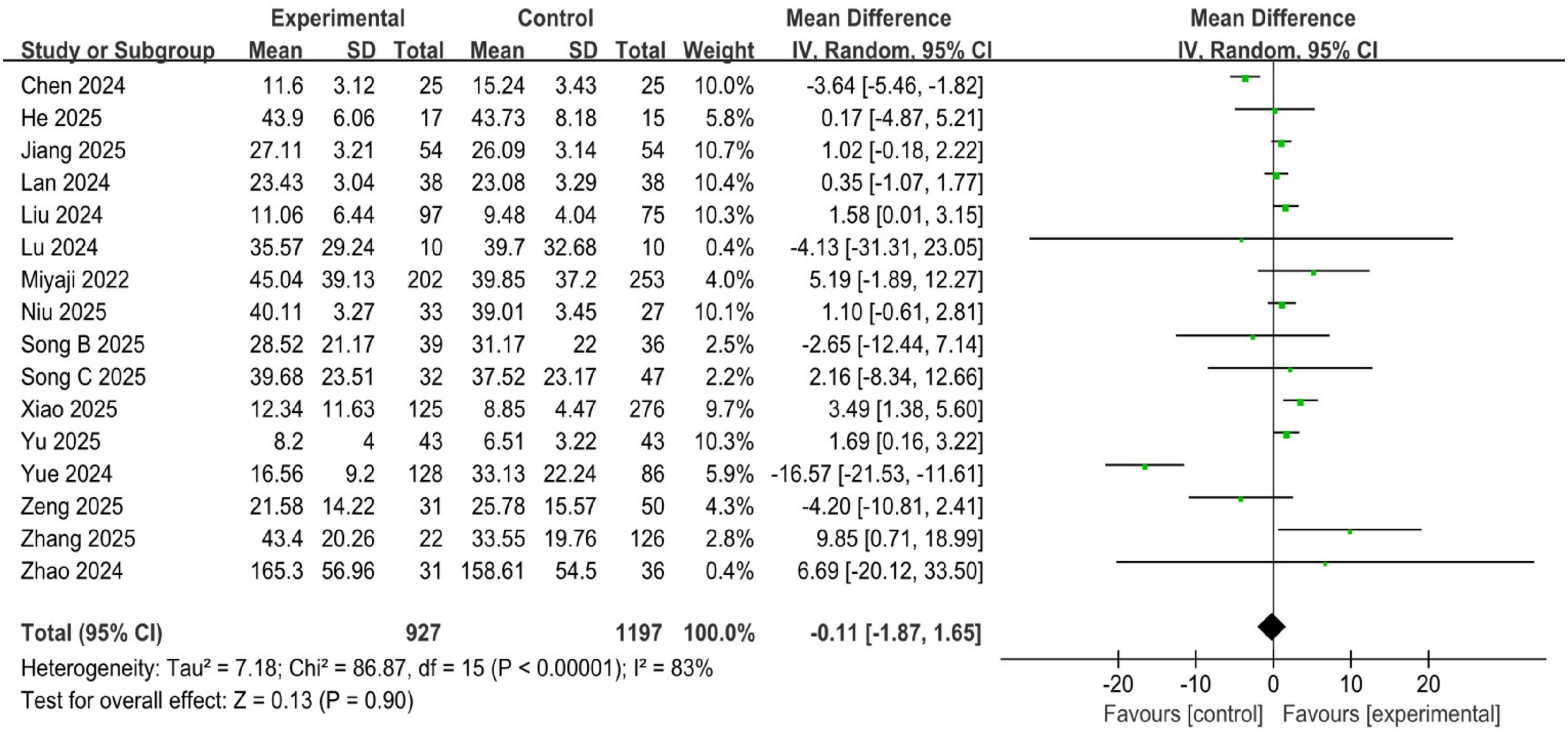
Forest plots of outcomes in filter lifespan.

A total of 16 studies (19–28, 31–36) reported filter lifespan, involving 2,124 CRRT patient episodes. There was statistical heterogeneity (*P* < 0.001, *I*^2^ = 83%), so we used the random-effects model. Results showed no significant difference in filter lifespan (h) between NM and citrate (MD = −0.11, 95%CI: −1.87 to 1.65, *p* = 0.90; Figure 3). We also conducted subgroup analyses to assess the effects of different bleeding risks, NM dosages, and study designs. Results showed no significant differences in filter lifespan across bleeding risks and NM dosages subgroups (*P* > 0.05). In the RCTs subgroup, NM showed a longer filter lifespan than citrate (MD = 0.97, 95%CI: 0.18 to 1.75, *p* = 0.02). Subgroup analyses are detailed in Table 2.

**TABLE 2:** Subgroup analyses of the effect of NM and citrate in filter lifespan.

Subgroup	Studies	Sample	Heterogeneity test		Effect model	Effect sizes			
			*I*^2^(%)	*P*		MD	95%CI	*Z*	*P*
Bleeding risks									
High-risk bleeding	8	618	72	<0.001	Random	0.21	−1.42∼1.84	0.25	0.80
Non-high-risk bleeding	8	1,506	89	<0.001	Random	−0.38	−4.59∼3.84	0.18	0.86
NM dosages									
Low-dose	10	1,172	84	<0.001	Random	−0.97	−3.36∼1.42	0.79	0.43
High-dose	6	952	82	<0.001	Random	1.18	−1.80∼4.17	0.78	0.44
Study designs									
RCTs	4	345	0	0.55	Fixed	0.97	0.18∼1.75	2.42	0.02
Observational studies	12	1,779	87	<0.001	Random	−0.51	−3.46∼2.44	0.34	0.73

#### 3.4.2 Bleeding events

**FIGURE 4:**
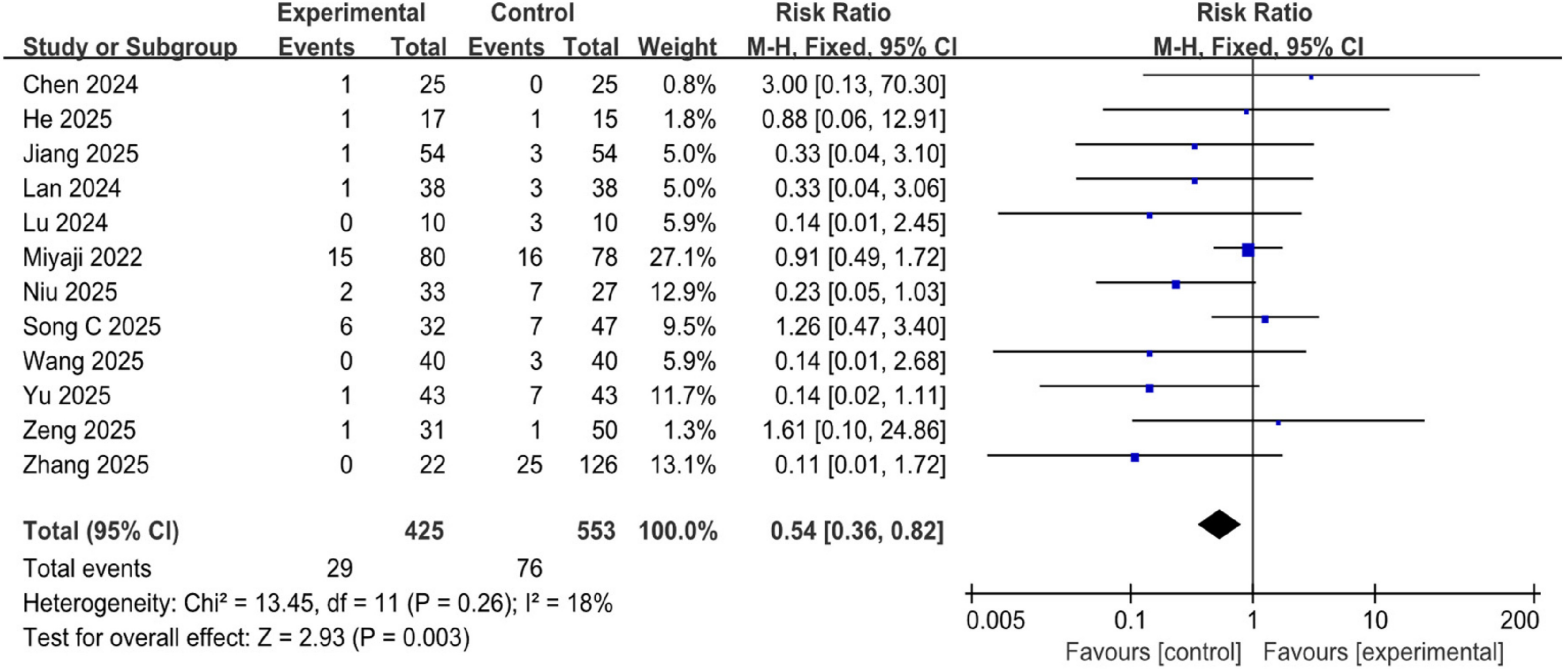
Forest plots of outcomes in bleeding events.

A total of 12 studies (19–22, 24–26, 28, 30, 32, 34, 35) reported bleeding events, involving 978 CRRT patient episodes. The analysis of data from the fixed-effect model showed that NM reduced the risk of bleeding events compared with citrate (RR = 0.54, 95%CI: 0.36–0.82, *p* = 0.003; Figure 4), with low heterogeneity (*p* = 0.26, *I*^2^ = 18%). Subgroup analyses showed that NM was associated with a lower risk of bleeding events in the high-risk bleeding, low-dose NM, and RCTs subgroups (RR = 0.47, 95%CI: 0.25–0.88, *p* = 0.02; RR = 0.39, 95%CI: 0.23–0.66, *p* < 0.001; RR = 0.21, 95%CI: 0.07–0.66, *p* = 0.007; Table 3).

**TABLE 3:** Subgroup analyses of the effect of NM and citrate in bleeding events.

Subgroup	Studies	Sample	Heterogeneity test		Effect model	Effect sizes			
			*I*^2^(%)	*P*		RR	95%CI	*Z*	*P*
Bleeding risks									
High-risk bleeding	6	371	37	0.16	Fixed	0.47	0.25∼0.88	2.37	0.02
Non-high-risk bleeding	6	607	0	0.42	Fixed	0.60	0.34∼1.04	1.83	0.07
NM dosages									
Low-dose	7	581	30	0.20	Fixed	0.39	0.23∼0.66	3.54	<0.001
High-dose	5	407	36	0.18	Fixed	0.64	0.30∼1.37	1.15	0.25
Study designs									
RCTs	4	350	0	0.91	Fixed	0.21	0.07∼0.66	2.68	0.007
Observational studies	8	628	18	0.29	Fixed	0.67	0.42∼1.04	1.77	0.08

#### 3.4.3 Clotting events

**FIGURE 5:**
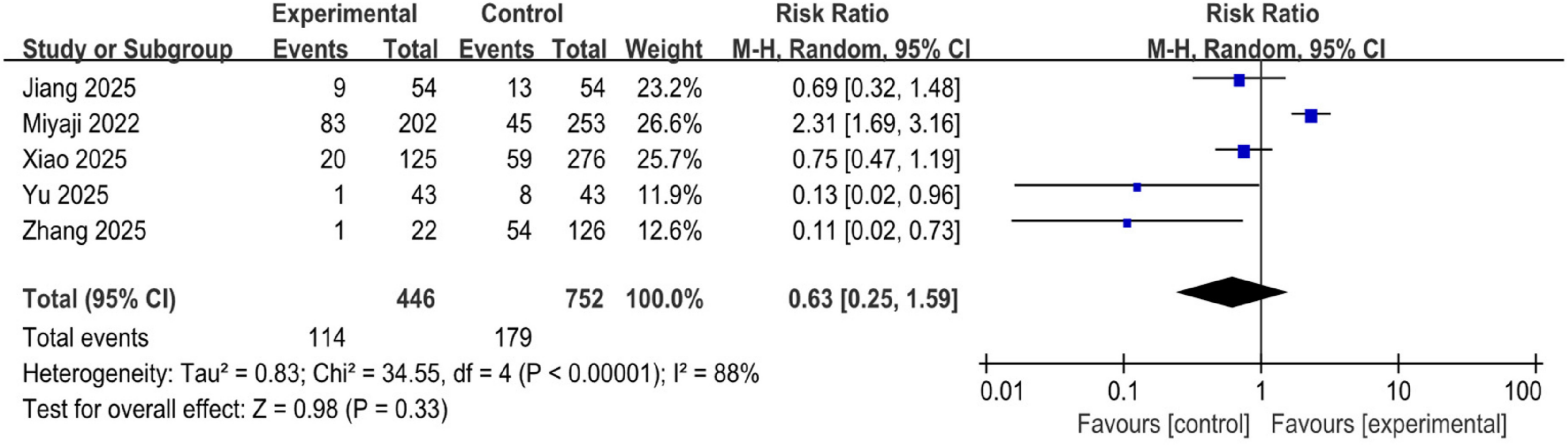
Forest plots of outcomes in clotting events.

A total of five studies (21, 25, 31, 32, 35) reported clotting events, involving 1,198 CRRT patient episodes. There was statistical heterogeneity (*P* < 0.001, *I*^2^ = 88%), so we used the random-effects model. Results showed no significant difference in clotting events between NM and citrate (RR = 0.63, 95%CI: 0.25–1.59, *p* = 0.33; Figure 5).

#### 3.4.4 PLT

**FIGURE 6:**
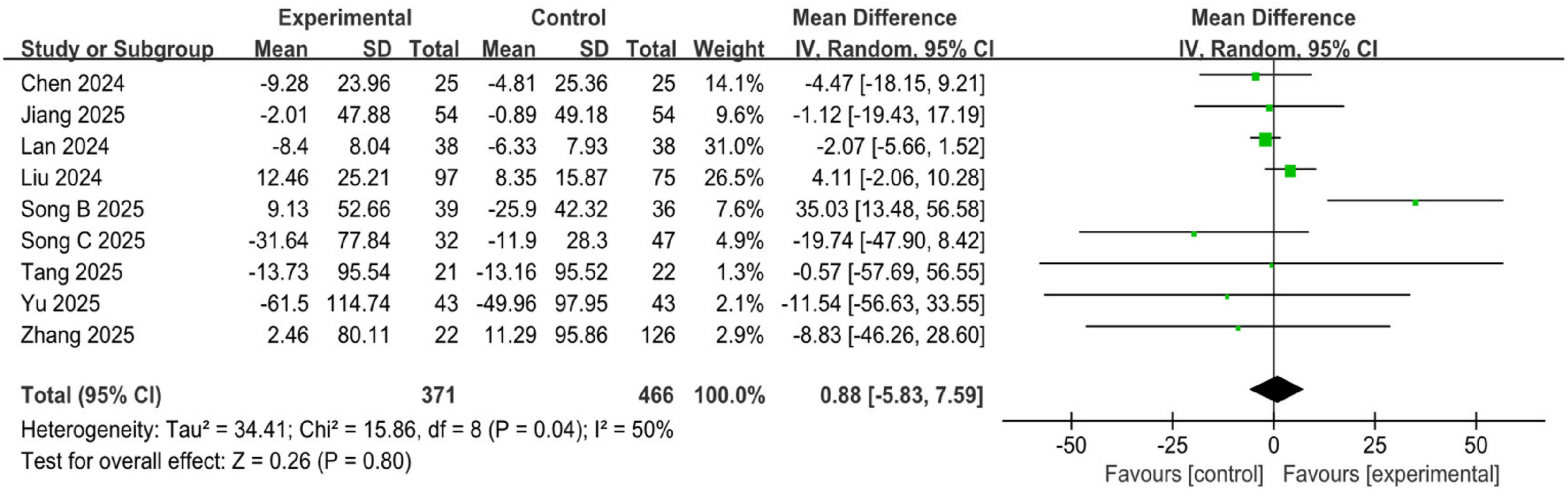
Forest plots of outcomes in PLT.

A total of 9 studies (19, 21–23, 27–29, 32, 35) reported platelet (PLT) counts, involving 837 CRRT patient episodes. There was statistical heterogeneity (*P* = 0.04, *I*^2^ = 50%), so we used the random-effects model. Results showed no significant difference in PLT between NM and citrate (MD = 0.88, 95%CI: −5.83 to 7.59, *p* = 0.80; Figure 6).

#### 3.4.5 APTT

**FIGURE 7:**
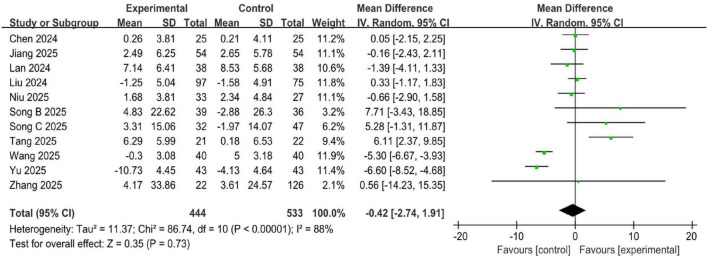
Forest plots of outcomes in APTT.

A total of 11 studies (19, 21–23, 26–30, 32, 35) reported activated partial thromboplastin time (APTT), involving 977 CRRT patient episodes. There was statistical heterogeneity (*P* < 0.001, *I*^2^ = 88%), so we used the random-effects model. Results showed no significant difference in APTT between NM and citrate (MD = −0.42, 95%CI: −2.74 to 1.91, *p* = 0.73; Figure 7).

#### 3.4.6 PT

**FIGURE 8:**
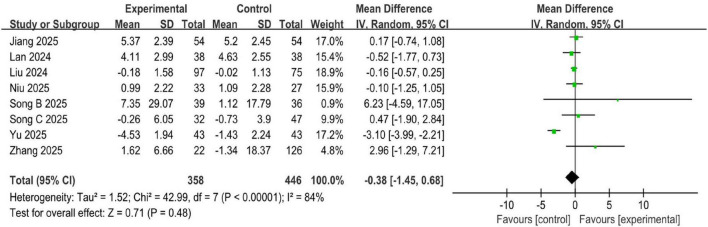
Forest plots of outcomes in PT.

A total of 8 studies (21–23, 26–28, 32, 35) reported prothrombin time (PT), involving 804 CRRT patient episodes. There was statistical heterogeneity (*P* < 0.001, *I*^2^ = 84%), so we used the random-effects model. Results showed no significant difference in PT between NM and citrate (MD = −0.38, 95%CI: −1.45 to 0.68, *p* = 0.48; Figure 8). After excluding (27), the *I*^2^-value decreased from 84 to 0%; the study subjects were patients with coagulation dysfunction, indicating that the heterogeneity originated in this study. The analysis of data from the fixed-effect model showed no significant difference in PT between NM and citrate (MD = −0.10, 95%CI: −0.43 to 0.24, *p* = 0.56).

### 3.5 Sensitive analysis

**FIGURE 9:**
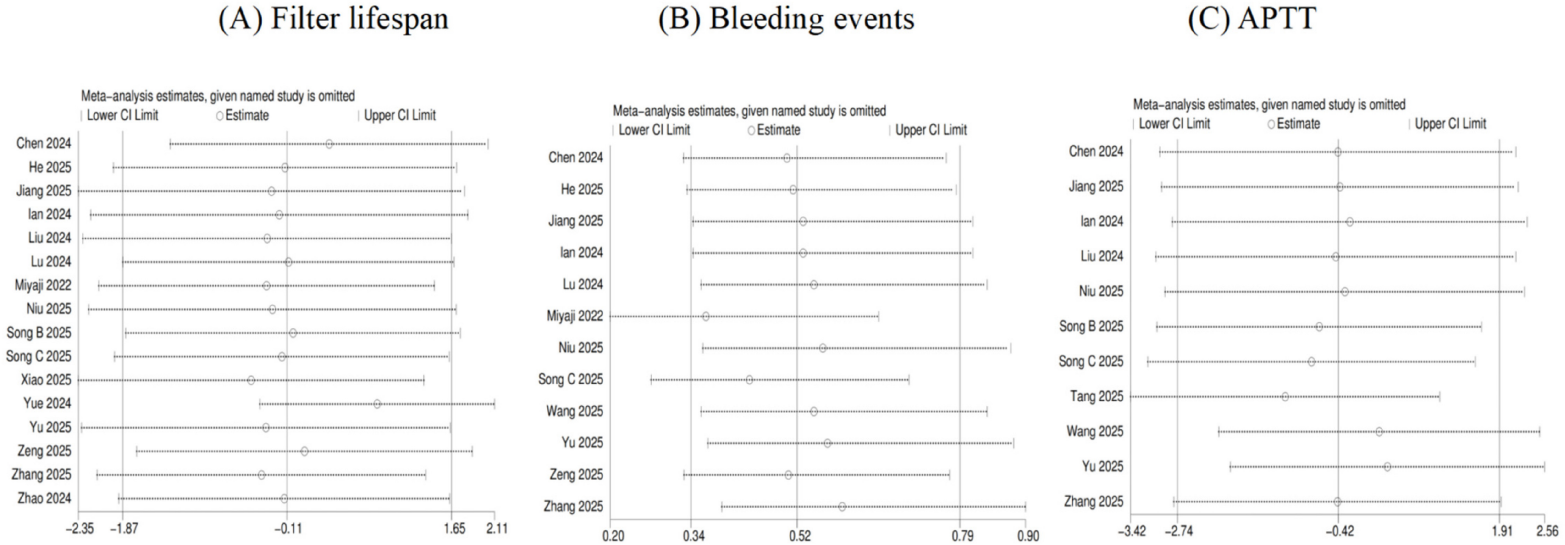
Sensitive analysis of outcomes in **(A)** filter lifespan; **(B)** bleeding events; **(C)** APTT.

Since sufficient studies evaluating filter lifespan, bleeding events, and APTT were available, we conducted leave-one-out sensitivity analyses by omitting 1 study at a time and recalculating estimates on the remaining studies. The results showed unchanged statistical conclusions. Hence, the meta-analysis results are stable. The results are detailed in Figure 9.

### 3.6 Publication bias

**FIGURE 10:**
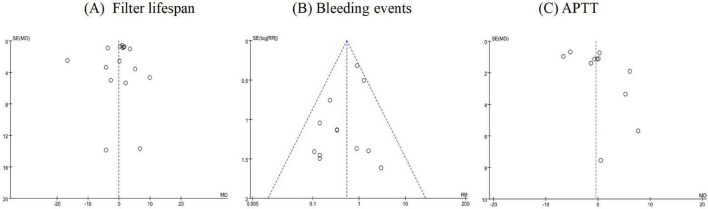
Funnel plot of outcomes in **(A)** filter lifespan; **(B)** bleeding events; **(C)** APTT.

We evaluated publication bias for filter lifespan, bleeding events, and APTT with funnel plots, which showed good symmetry. To further validate this, we conducted Egger’s tests. No evidence of substantial publication bias existed in this meta-analysis on filter lifespan (*t* = −0.57, *p* = 0.576), bleeding events (*t* = −1.97, *p* = 0.077), and APTT (*t* = 1.63, *p* = 0.138). Funnel plots are detailed in Figure 10.

### 3.7 Quality of evidence assessment (GRADE)

Due to the predominance of observational studies and the poor quality of RCTs, the initial rating for outcomes began at the “low” level. The overall quality of evidence ranged from “low” to “very low.” Five outcomes were downgraded for statistical heterogeneity, and insignificant results also contributed to the low quality. A summary of GRADE assessments is detailed in Supplementary Document 4.

## 4 Discussion

NM is the primary anticoagulant for blood purification in Japan. In contrast, RCA remains predominant in Europe and North America, with limited research on NM. Building on the widespread adoption of RCA, China introduced NM for CRRT anticoagulation in 2020 and subsequently conducted comparative studies of the two regimens. However, existing research remains fragmented and inconclusive. This systematic review and meta-analysis included 18 studies involving 2,247 CRRT patient episodes. The results indicate that compared with citrate, NM showed no significant differences in filter lifespan or clotting events (MD = −0.11, 95%CI: −1.87 to 1.65, *p* = 0.90; RR = 0.63, 95%CI: 0.25–1.59, *p* = 0.33). NM significantly reduced the risk of bleeding events (RR = 0.54, 95%CI: 0.36–0.82, *p* = 0.003), subgroup analyses showed that NM was associated with a lower risk of bleeding events in high-risk bleeding, low-dose NM, and RCTs subgroups (RR = 0.47, 95%CI: 0.25–0.88, *p* = 0.02; RR = 0.39, 95%CI: 0.23–0.66, *p* < 0.001; RR = 0.21, 95%CI: 0.07–0.66, *p* = 0.007), NM may serve as an alternative to RCA. China issued an expert consensus on the application of nafamostat mesylate for anticoagulation during blood purification in 2024 (37), recommending its use for patients with high bleeding risk or active bleeding, and confirming its clinical safety and effectiveness.

This study found no statistically significant differences in filter lifespan and clotting events between NM and citrate, suggesting comparable anticoagulant efficacy between the two regimens. This aligns with the conclusions of the systematic review by Liu et al. (10). Citrate binds to free calcium ions (coagulation factor IV) in the circulating blood, forming calcium citrate chelates that block the coagulation cascade reaction (38). NM exerts its anticoagulant effects by inhibiting thrombin, coagulation factors (VIIa, XIIa, and Xa), the kallikrein-kinin system, and the complement system (39). It also inhibits platelet aggregation by suppressing the secretion of arachidonic acids (including phospholipase A2) and promotes the disaggregation of aggregated platelets (40, 41). Despite differing mechanisms of action, both ultimately effectively inhibit fibrin formation, the key step in CRRT-associated coagulation. At standard clinical doses, both agents achieve adequate anticoagulation; differences in mechanism no longer determine filter lifespan, and thus both demonstrate comparable anticoagulant efficacy. The KDIGO clinical practice guidelines (2) recommend RCA as the preferred approach for CRRT. However, clinical practice often presents contraindications to the use of citrate. Citrate may affect electrolyte metabolism, leading to complications such as citrate acidosis, metabolic alkalosis, and hypocalcaemia. It necessitates frequent monitoring, involves complex procedures, and demands a high level of professional competence from healthcare personnel (42). In contrast, NM has no absolute contraindications. It exerts minimal effects on hepatic function and the internal environment, and may serve as an alternative to RCA. Clinicians may formulate individualized anticoagulation regimens based on patient-specific circumstances. Dissolve the NM powder in 5% glucose injection, then add 0.9% sodium chloride injection to prepare the priming solution, achieving a concentration of 20–40 mg/L. Dissolve and prepare the NM infusion solution using 5% glucose injection. Administer via continuous intravenous infusion concurrently with the initiation of extracorporeal circulation. Adjust the dosage based on monitoring results of activated clotting time (ACT) or APTT (43, 44). When using polyacrylonitrile membranes with high NM adsorption capacity, such as AN69 or AN69ST, the drug dose must be correspondingly increased to achieve equivalent, safe, and individualized CRRT anticoagulation management (45).

This study found that a total of 13 studies measured and reported bleeding events. However, 1 study (27) reported no bleeding events in either group, making it impossible to calculate RR and 95%CI. Therefore, only 12 studies were included in the meta-analysis. The meta-analysis found that NM significantly reduced the risk of bleeding events in CRRT patients compared with citrate. This conclusion is inconsistent with the systematic review findings of Liu et al. (10) and Lin et al. (9), both of which included only the single-center clinical study by Uchino et al. (46), which reported 2 bleeding events in each group, limiting statistical power and generalizability. Calcium citrate chelate undergoes hepatic metabolism via the tricarboxylic acid cycle, forming bicarbonate and calcium ions. Its half-life is 18–54 min, necessitating exogenous calcium supplementation. If patients exhibit hepatic impairment or hypoxaemia, calcium citrate metabolism may be affected. This can lead to citrate accumulation and fluctuations in calcium levels, thereby increasing the risk of bleeding (37, 47). In contrast, NM has a molecular weight of only 539.58 Da, below the retention threshold of conventional filters. Approximately 40% is removed by filters, with less than 4% entering the body (48). NM is rapidly metabolized via both hepatic and blood pathways, with a half-life of only 5–8 min, and does not accumulate even in hepatic impairment (5). Systemic adverse reactions are less frequent than with other anticoagulants, correspondingly reducing bleeding risk. Consequently, in clinical practice, NM may be a relatively safe anticoagulant regimen that does not rely on hepatic metabolism or calcium ion stability.

Building on these findings, this study further conducted subgroup analyses of filter lifespan and bleeding events. This assessed the impact of varying bleeding risks, NM dosages, and study designs. In the high-risk bleeding subgroup, both NM and citrate demonstrated their respective anticoagulant effects, with comparable filter lifespans. However, NM had a lower risk of bleeding events. High bleeding risk patients frequently present with prolonged clotting times, thrombocytopenia, recent active bleeding, trauma or surgery history, and diminished systemic coagulation reserves. Impairment of calcium citrate metabolism predisposes these patients to systemic hypocalcaemia, which further compromises coagulation function and heightens bleeding risk. In contrast, the anticoagulant mechanism of NM does not involve coagulation factor IV and is unaffected by fluctuations in calcium ion levels (23). The expert consensus (37) defines a NM dose of ≤ 30 mg/h as the low-dose group. The clinical study by Kameda et al. (49) confirmed that doses below 30 mg/h ensure adequate filter lifespan and reduce systemic exposure with a favorable safety profile. Consequently, we exploratively classified doses ≤ 30 mg/h as a low-dose subgroup; the results suggest that anticoagulant efficacy in the low-dose NM subgroup is comparable to that of citrate. Consequently, NM may offer superior clinical utility for CRRT patients at high bleeding risk. For patients with hypercoagulable states or requiring heterogeneous mode therapy, individualized dose escalation of NM is warranted. In the RCTs subgroup, although the difference in filter lifespan was statistically significant, the magnitude was small (MD = 0.97, *p* = 0.02) and of limited clinical significance. Furthermore, this subgroup included only 4 studies with low methodological quality. Consequently, the conclusions regarding filter lifespan and bleeding risk for nimesulide in the RCTs subgroup should be regarded as exploratory findings; the results should be interpreted with caution. Further high-quality, large-scale RCTs are required to validate these findings.

None of the 18 included studies reported serious adverse reactions associated with NM, including eosinophilia, agranulocytosis, bone marrow suppression, or severe allergic reactions. Hyperkalemia was reported in only 2 studies: Song et al. (28) documented 2 cases in the NM group and 1 in the citrate group. Zeng et al. (34) reported 2 and 3 cases, respectively, with no statistically significant difference between groups. Hyperkalemia can be effectively prevented and managed through precise solute control techniques. 1 additional study (26) documented mild allergic reactions: the NM group experienced 2 cases of nausea, 1 case of rash, and 2 cases of mild fever; the citrate group reported 1 case of nausea, 1 case of vomiting, and 1 case of local phlebitis. All recovered following symptomatic management. Although the incidence of adverse reactions to NM is low and its overall safety profile is favorable, clinical practice requires vigilant monitoring for occasional allergic reactions such as nausea, vomiting, rash, and fever. Where necessary, rapid assessment via skin reaction testing may be employed (50). From an economic perspective, (25) reported that the cost of anticoagulation with generic NM in Japan was approximately 1/3 that of citrate (daily cost $154 vs. $527). NM was formally included in China’s National Essential Medicines List in 2022, further reducing patient burden and enhancing its clinical accessibility. However, NM costs vary across countries due to differing economic conditions and policies.

In summary, this study suggested that NM may be feasible as an anticoagulant in CRRT, with anticoagulant efficacy comparable to citrate and good safety. In clinical practice, NM may serve as a viable anticoagulant alternative for CRRT patients with contraindications to citrate or high bleeding risk. This study has the following limitations: the included literature predominantly comprised observational studies, with only 5 RCTs; the overall certainty of the evidence is low to very low, which limits the strength of the conclusions; the findings should be interpreted with caution. Only Chinese and English literature was included, with no Japanese literature; furthermore, as NM is not yet widely used in Europe and North America, there is a risk of systematically overestimating efficacy or underestimating the risk of adverse events due to language bias and geographic bias, thereby affecting the generalizability of the findings. The definitions of bleeding events across the original studies were not entirely consistent, which may introduce some bias. This study did not explicitly exclude patients with contraindications to citrate, which may affect the relevance of the findings to the target treatment population. The included studies differed in patient characteristics, filter types, CRRT modalities, blood flow rates, priming methods, and drug doses, which may have influenced the pooled analysis results. As some outcome measures exhibited high heterogeneity, and due to missing information in the original studies and sample size limitations, meta-regression was not performed to further identify the sources of heterogeneity. Further research should focus on high-quality RCTs, especially for pediatric, citrate-contraindicated, or extracorporeal membrane oxygenation (ECMO) patients, prioritizing patient-centered outcomes. Future studies should include searches of Japanese databases or conduct multicenter international trials, thereby providing more robust evidence to support the development of individualized anticoagulation regimens.

## Data Availability

The original contributions presented in the study are included in the article/Supplementary material, further inquiries can be directed to the corresponding author.
