# Supplementary material for: Comparing the efficacy and safety of nafamostat mesylate versus citrate for anticoagulation in continuous renal replacement therapy: a systematic review and meta-analysis
Source: Front Med (Lausanne). 2026 Jul 6;13:1831023. doi: 10.3389/fmed.2026.1831023 (PMC13381840; doi:10.3389/fmed.2026.1831023)
Supplement: Supplementary file 2 [file Table_2.DOCX]

Supplementary Document 2: Search strategy

1. PubMed

| Search ID | Search formula |
| --- | --- |
| #1 | "nafamostat"[Title/Abstract] OR "nafamostat mesylate"[Title/Abstract] OR "nafamostat mesilate"[Title/Abstract] OR "nafamostat mediate"[Title/Abstract] OR "NM"[Title/Abstract] OR "nafamostat dimethanesulfonate"[Title/Abstract] OR "nafamostat dihydrochloride"[Title/Abstract] OR "futhan"[Title/Abstract] OR "6'-amidino-2-naphthyl 4-guanidinobenzoate"[Title/Abstract] OR "FUT-175"[Title/Abstract] OR "CKD-314"[Title/Abstract] |
| #2 | "Continuous Renal Replacement Therapy"[Mesh] |
| #3 | "CRRT"[Title/Abstract] OR "continuous kidney replacement therapy"[Title/Abstract] OR "CKRT"[Title/Abstract] OR "blood purification"[Title/Abstract] OR "hemopurification"[Title/Abstract] OR "continuous venovenous hemofiltration"[Title/Abstract] OR "CVVH"[Title/Abstract] OR "continuous venovenous hemodialysis"[Title/Abstract] OR "CVVHD"[Title/Abstract] OR "continuous venovenous hemodiafiltration"[Title/Abstract] OR "CVVHDF"[Title/Abstract] OR "slow continuous ultrafiltration"[Title/Abstract] OR "SCUF"[Title/Abstract] |
| #4 | #2 or #3 |
| #5 | #1 and #4 |

1. Embase

| Search ID | Search formula |
| --- | --- |
| #1 | 'nafamostat'/exp OR ‘nafamostat mesylate’:ti,ab,kw OR ‘nafamostat mesilate’:ti,ab,kw OR ‘nafamostat mediate’:ti,ab,kw OR ‘NM’:ti,ab,kw OR ‘nafamostat dimethanesulfonate’:ti,ab,kw OR ‘nafamostat dihydrochloride’:ti,ab,kw OR ‘futhan’:ti,ab,kw OR ‘6'-amidino-2-naphthyl 4-guanidinobenzoate’:ti,ab,kw OR ‘FUT-175’:ti,ab,kw OR ‘CKD-314’:ti,ab,kw |
| #2 | 'continuous renal replacement therapy'/exp OR ‘CRRT’:ti,ab,kw OR ‘continuous kidney replacement therapy’:ti,ab,kw OR ‘CKRT’:ti,ab,kw OR ‘blood purification’:ti,ab,kw OR ‘hemopurification’:ti,ab,kw OR ‘continuous venovenous hemofiltration’:ti,ab,kw OR ‘CVVH’:ti,ab,kw OR ‘continuous venovenous hemodialysis’:ti,ab,kw OR ‘CVVHD’:ti,ab,kw OR ‘continuous venovenous hemodiafiltration’:ti,ab,kw OR ‘CVVHDF’:ti,ab,kw OR ‘slow continuous ultrafiltration’:ti,ab,kw OR ‘SCUF’:ti,ab,kw |
| #3 | #1 and #2 |

1. Web of science

| Search ID | Search formula |
| --- | --- |
| #1 | TS= (nafamostat OR nafamostat mesylate OR nafamostat mesilate OR nafamostat mediate OR NM OR nafamostat dimethanesulfonate OR nafamostat dihydrochloride OR futhan OR 6'-amidino-2-naphthyl 4-guanidinobenzoate OR FUT-175 OR CKD-314) |
| #2 | TS= (continuous renal replacement therapy OR CRRT OR continuous kidney replacement therapy OR CKRT OR blood purification OR hemopurification OR continuous venovenous hemofiltration OR CVVH OR continuous venovenous hemodialysis OR CVVHD OR continuous venovenous hemodiafiltration OR CVVHDF OR slow continuous ultrafiltration OR SCUF) |
| #3 | #1 and #2 |

1. Cochrane

| Search ID | Search formula |
| --- | --- |
| #1 | (nafamostat):ti,ab,kw OR (nafamostat mesylate):ti,ab,kw OR (nafamostat mesilate):ti,ab,kw OR (nafamostat mediate):ti,ab,kw OR (NM):ti,ab,kw OR (nafamostat dimethanesulfonate):ti,ab,kw OR (nafamostat dihydrochloride):ti,ab,kw OR (futhan):ti,ab,kw OR (6'-amidino-2-naphthyl 4-guanidinobenzoate):ti,ab,kw  OR (FUT-175):ti,ab,kw OR (CKD-314):ti,ab,kw |
| #2 | MeSH descriptor: [Continuous Renal Replacement Therapy] explode all trees |
| #3 | (CRRT):ti,ab,kw OR (continuous kidney replacement therapy):ti,ab,kw OR (CKRT):ti,ab,kw OR (blood purification):ti,ab,kw OR (hemopurification):ti,ab,kw |
| #4 | (continuous venovenous hemofiltration):ti,ab,kw OR (CVVH):ti,ab,kw OR (continuous venovenous hemodialysis):ti,ab,kw OR (CVVHD):ti,ab,kw |
| #5 | (continuous venovenous hemodiafiltration):ti,ab,kw OR (CVVHDF):ti,ab,kw OR (slow continuous ultrafiltration):ti,ab,kw OR (SCUF):ti,ab,kw |
| #6 | #2 or #3 or #4 or #5 |
| #7 | #1 and #6 |

1. CBM

| Search ID | Search formula |
| --- | --- |
| #1 | 摘要= (萘莫司他 OR 甲磺酸萘莫司他 OR 甲磺酸酯奈莫司他 OR 6 '-脒基-2-萘基4-胍基苯甲酸酯) |
| #2 | 主题词=连续性肾替代疗法 |
| #3 | 摘要= (连续性肾脏替代治疗 OR CRRT OR 血液净化 OR 连续性静脉-静脉血液透析 OR 连续性静脉-静脉血液滤过 OR 连续性静脉-静脉血液透析滤过 OR 缓慢连续性超滤) |
| #4 | #2 or #3 |
| #5 | #1 and #4 |

1. CNKI

| Search ID | Search formula |
| --- | --- |
| #1 | 主题= (萘莫司他 OR 甲磺酸萘莫司他 OR 甲磺酸酯奈莫司他 OR 6 '-脒基-2-萘基4-胍基苯甲酸酯) |
| #2 | 主题= (连续性肾脏替代治疗 OR CRRT OR 血液净化 OR 连续性静脉-静脉血液透析 OR 连续性静脉-静脉血液滤过 OR 连续性静脉-静脉血液透析滤过 OR 缓慢连续性超滤) |
| #3 | #1 and #2 |

1. Wanfang

| Search ID | Search formula |
| --- | --- |
| #1 | 主题= (萘莫司他 OR 甲磺酸萘莫司他 OR 甲磺酸酯奈莫司他 OR 6 '-脒基-2-萘基4-胍基苯甲酸酯) |
| #2 | 主题= (连续性肾脏替代治疗 OR CRRT OR 血液净化 OR 连续性静脉-静脉血液透析 OR 连续性静脉-静脉血液滤过 OR 连续性静脉-静脉血液透析滤过 OR 缓慢连续性超滤) |
| #3 | #1 and #2 |

1. VIP

| Search ID | Search formula |
| --- | --- |
| #1 | 篇关摘= (萘莫司他 OR 甲磺酸萘莫司他 OR 甲磺酸酯奈莫司他 OR 6 '-脒基-2-萘基4-胍基苯甲酸酯) |
| #2 | 篇关摘= (连续性肾脏替代治疗 OR CRRT OR 血液净化 OR 连续性静脉-静脉血液透析 OR 连续性静脉-静脉血液滤过 OR 连续性静脉-静脉血液透析滤过 OR 缓慢连续性超滤) |
| #3 | #1 and #2 |
