# Supplementary material for: Comparing the efficacy and safety of nafamostat mesylate versus citrate for anticoagulation in continuous renal replacement therapy: a systematic review and meta-analysis
Source: Front Med (Lausanne). 2026 Jul 6;13:1831023. doi: 10.3389/fmed.2026.1831023 (PMC13381840; doi:10.3389/fmed.2026.1831023)
Supplement: Supplementary file 3 [file Table_3.DOCX]

Supplementary Document 3: Evaluation of risk of bias for observational studies by the ROBINS-I tool

| Study | confounding | selection of participants | intervention | deviations from intended interventions | missing data | measurement of outcomes | selection of the reported result | Overall |
| --- | --- | --- | --- | --- | --- | --- | --- | --- |
| Chen et al. (2024) | serious | low | moderate | low | low | moderate | moderate | Serious |
| He et al. (2025) | moderate | low | moderate | low | low | moderate | moderate | Moderate |
| Liu et al. (2024) | moderate | low | moderate | low | low | low | moderate | Moderate |
| Lu et al. (2024) | serious | low | moderate | low | low | low | serious | Serious |
| Miyaji et al. (2022) | moderate | moderate | moderate | low | low | low | moderate | Moderate |
| Niu et al. (2025) | serious | low | moderate | low | low | low | moderate | Serious |
| Song C et al. (2025) | moderate | low | moderate | low | low | low | moderate | Moderate |
| Tang et al. (2025) | serious | moderate | moderate | low | low | low | serious | Serious |
| Xiao et al. (2025) | moderate | moderate | low | low | moderate | low | moderate | Moderate |
| Zeng et al. (2025) | moderate | low | moderate | low | low | low | moderate | Moderate |
| Zhang et al. (2025) | serious | low | moderate | low | low | low | serious | Serious |
| Zhao et al. (2024) | serious | low | moderate | low | low | moderate | moderate | Serious |
| Yue et al. (2024) | moderate | low | moderate | low | low | low | moderate | Moderate |

Abbreviations: ROBINS-I=Risk Of Bias In Non-randomised Studies-of Interventions.
