# Supplementary material for: Comparing the efficacy and safety of nafamostat mesylate versus citrate for anticoagulation in continuous renal replacement therapy: a systematic review and meta-analysis
Source: Front Med (Lausanne). 2026 Jul 6;13:1831023. doi: 10.3389/fmed.2026.1831023 (PMC13381840; doi:10.3389/fmed.2026.1831023)
Supplement: Supplementary file 4 [file Table_4.DOCX]

Supplementary Document 4: Quality of evidence

| **Outcomes** | **Number of studies** | **Design** | **Downgrading factors** | | | | | **Upgrading factors** | | | **GRADE** |
| --- | --- | --- | --- | --- | --- | --- | --- | --- | --- | --- | --- |
|  |  |  | **Risk of bias** | **Inconsistency** | **Indirectness** | **Imprecision** | **Publication bias** | **Large Magnitude of Effect** | **Dose-Response Gradient** | **All Plausible Confounding Would Reduce the Effect** |  |
| Filter lifespan | 16 | 4 RCTs  12 observational studies | serious | serious^a^ | not serious | serious^b^ | none | none | none | none | ⨁◯◯◯ Very low |
| Clotting events | 5 | 2 RCT  3 observational studies | not serious | serious^a^ | not serious | serious^b^ | none | none | none | none | ⨁◯◯◯ Very low |
| Bleeding events | 12 | 4 RCT  8 observational studies | not serious | not serious | not serious | not serious | none | none | none | none | ⨁⨁◯◯ Low |
| PLT | 9 | 4 RCTs  5 observational studies | serious | serious^a^ | not serious | serious^b^ | none | none | none | none | ⨁◯◯◯ Very low |
| APTT | 11 | 5 RCTs  6 observational studies | serious | serious^a^ | not serious | serious^b^ | none | none | none | none | ⨁◯◯◯ Very low |
| PT | 8 | 4 RCTs  4 observational studies | serious | serious^a^ | not serious | serious^b^ | none | none | none | none | ⨁◯◯◯ Very low |

Abbreviations: PLT=platelet; APTT=activated partial thromboplastin time; PT=prothrombin time; RCT=randomised controlled trial; GRADE=Grading of Recommendations Assessment, Development, and Evaluation.

Explanations:

a=Downgrading one level for the heterogeneity ≥ 50%.

b=Downgrading one level for the effect size not significant.
